# Analysis of Muscle Oxygenation after a Normobaric Hypoxia Tolerance Test

**DOI:** 10.3390/jfmk9020086

**Published:** 2024-05-14

**Authors:** Inés Albertus-Cámara, María-José Paredes-Ruiz, Ignacio Martínez-González-Moro

**Affiliations:** Physical Exercise and Human Performance Research Group, Mare Nostrum Campus, University of Murcia, 30001 Murcia, Spain; ines.albertusc@gmail.com (I.A.-C.); mariajoseparedesruiz@gmail.com (M.-J.P.-R.)

**Keywords:** normobaric hypoxia, muscle oxygen saturation, near-infrared spectrometry

## Abstract

The aim of this work was to analyze the influence of acute normobaric hypoxia on quadricep oxygenation. Muscle oxygen saturation (SmO_2_) was measured using near-infrared spectrometry (NIRS) technology during a normobaric hypoxia tolerance test (NHTT). SmO_2_ was measured with a Humon Hex^®^ device. In total, 54 healthy subjects participated, 68.5 of which were males and 31.5% of which were females. They performed an NHTT with the IAltitude^®^ simulator, breathing air with an FiO_2_ level of 11% (equivalent to 5050 m). The maximum duration of the NHTT was set at 10 min, stopping if it reached 83% SpO_2_. The initial values (PRE) were compared with those obtained at the end of the test (POST) and after 10 min of recovery. The participants were divided into two groups based on whether (G1) they completed the ten minutes or not (G2). In total, 35.1% of men and 41.2% of women completed the 10 min. In both groups, significant differences were observed in the decrease in SmO_2_ values (*p* < 0.0001) (G1: PRE = 59.5 ± 12.48%; POST = 55.95 ± 14.30%; G2: PRE = 60.06 ± 13.46%; POST = 57.2 ± 12.3%). There were no differences between groups in any of the three periods. Exposure to normobaric hypoxia produces a decrease in quadricep levels of SmO_2_ in both sexes, regardless of whether the test is completed. Two patterns appeared: A.—less time and more hypoxia; B. a longer duration and less hypoxia.

## 1. Introduction

The use of physiological adaptations to altitude (increased hemoglobin and hematocrit), and therefore to hypoxia, is a classic procedure to improve the availability of oxygen (O_2_) in the blood, which has led to multiple studies on acclimatization and physical exercise at certain altitudes [1]. It is also associated with a training system known as “altitude training”. This system is performed in situations of hypobaric hypoxia and requires the athlete and their technical team to travel to specialized centers located at a significant altitude with respect to sea level. The athlete can live and train at a higher altitude or just live there and train in facilities at a lower altitude, depending on the procedure used in each situation [2]. Technological evolution has given rise to a technique called “normobaric hypoxia” (NH), in which devices are used to reduce and control the amount of oxygen in the inspired air, without the need to move the athlete to altitude centers and therefore without changing the atmospheric pressure [3].

Normobaric hypoxia is increasingly being used, not only as an aid to sports training, but also as a therapeutic resource [4]. In all cases, before performing hypoxia conditioning sessions, it is necessary to know the individual’s tolerance to hypoxia and with that information plan the sessions. Normobaric hypoxia tolerance tests (NHTTs) are performed to determine the impact of hypoxia on arterial oxygen saturation (SpO_2_) and heart rate (HR) [5]. NHTTs subject the individual to a low and constant inspired fraction of oxygen, FiO_2_, for a certain period, observing the decrease in SpO_2_ and the clinical response of the subject [6]. Thus, NHTTs end when the established time is fulfilled without reaching the safe SpO_2_ value or when the subject reaches the minimum agreed SpO_2_ value [7].

Carrying out tolerance tests before starting a training or rehabilitation program in normobaric hypoxia makes it possible to detect people who suffer acute decreases in SpO_2_, maintaining safety and adjusting the doses of hypoxia they will receive in training sessions [8]. In this way, the first sessions will be carried out with higher FiO_2_ figures and will be progressively decreased. On the other hand, in people with good tolerance, that is, with a lower decrease in SpO_2_ during NHTTs, sessions can be started with a lower concentration of oxygen, as if they were at a higher altitude. This allows participants to personalize their sessions [9].

For some years now, muscle oxygenation has been studied by determining muscle oxygen saturation (SmO_2_) [10]. One of the bloodless procedures is based on estimating the percentage of muscle saturation from the ratio of oxyhemoglobin to total hemoglobin in the blood, determined by near-infrared spectroscopy (NIRS) [11]. The absorption of infrared light varies depending on the state of oxygenation and deoxygenation of the hemoglobin and myoglobin molecules, allowing the percentage of oxygenation to be quantified [12,13]. NIRS-based technology can be considered an excellent technique with which to describe the acute changes produced by an exercise session, such as training-induced improvements in the oxidative system, and it can be applied to improve performance in physical sports activities [14].

Changes in SmO_2_ during exercise are related to the availability of oxygen in the blood and the use of that oxygen in active muscles [15]. It has been proven that a favorable rate of SmO_2_ is associated with greater physical exercise capacity [16]. Conversely, the decrease in oxygen in inspired air causes SpO_2_ to decrease. We consider it interesting to determine whether or not NIRS can be used to detect changes in SmO_2_ induced by an NHTT. This could be used to determine if SmO_2_ can be used as a more specific NH training planning and control element than SpO_2_ monitoring, maintaining control of SpO_2_ as a security measure and of SmO_2_ for training customization.

It is known that the response to hypoxia is independent of the person’s gender, and that adequate muscle oxygenation is necessary to optimize muscle strength and endurance. Therefore, we consider it interesting to determine the effect of normobaric hypoxia on SmO_2_. Our hypothesis is that NH induces changes in SmO_2_ and that these appear in both men and women. Therefore, the aim of this study is to determine whether or not performing a hypoxia tolerance test is a sufficient stimulus to generate changes in muscle oxygenation measured with near-infrared spectroscopy, determining the influence of gender and body composition on these changes.

## 2. Materials and Methods

### 2.1. Design

This study is a cross-sectional design that aims to establish the influence of acute exposure to normobaric hypoxia with an FiO_2_ percentage of 11%, equivalent to an altitude of 5050 m, on SmO_2_. To determine this, the SmO_2_ of the quadriceps was measured before and after a hypoxia tolerance test lasting 10 min or a decrease in SpO_2_ of less than 83%. This study was approved by the Bioethics Committee of the Murcia University (Spain) (ID: 4142/2022) and complies with the Declaration of Helsinki. All the participants signed the informed consent form.

**Figure 1:**
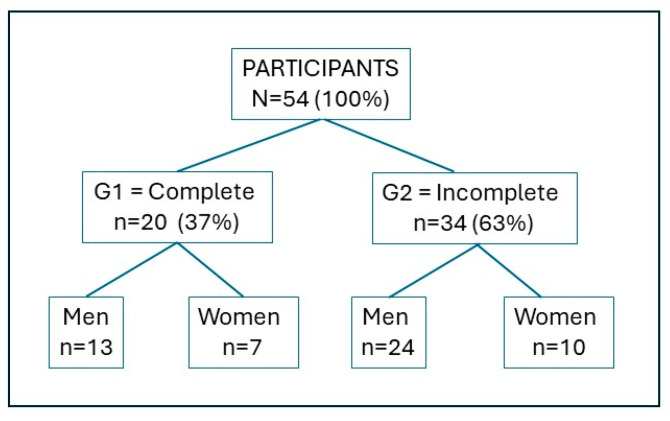
Participant flowchart.

Subsequently, the population was divided into two groups according to whether or not they had completed the maximum time of exposure to hypoxia (G1 = Complete = 10 min; G2 = Incomplete < 10 min), divided into males and females (Figure 1).

### 2.2. Participants

In total, 54 subjects were recruited, 37 of them male (68.5%), recreational athletes who came to the laboratory of our Research Group. The inclusion criteria were as follows: age between 18 and 55 years, and no acute pathologies at the time of the test. The exclusion criteria were as follows: presence of cardiorespiratory diseases that contraindicate the performance of NHTT, pregnancy, and hematological and metabolic alterations. Participants filled out and signed a questionnaire with their medical history based on their medical history. This was supervised by a specialist doctor who carried out the initial clinical assessment.

### 2.3. Procedure

The study was divided into five stages.

Firstly, participants completed the informed consent form and a questionnaire on sports medical history to rule out pathologies and obtaining anthropometric and body composition data including the following: weight, height, waist circumference, fat percentage and percentage of muscle by bioimpedance (Inbody 120, Inbody Co., Seoul, Republic of Korea). Body composition analysis was performed with participants standing, barefoot on the platform, and wearing light clothing during the measurement. They were instructed to fast for three hours before the tests, not to drink coffee, tea, or alcoholic beverages for 12 h prior the test, and to evacuate their bladder before the measurement.

Second: A cardiovascular examination was performed at rest, to rule out pathologies that may have worsened with NH, including an electrocardiogram (Clickecgbt, Cardioline, Cavareno, Italy) and echocardiography (Clarius PA HD3, Clarius, Burnaby, BC, Canada), by a specialist physician. A standard electrocardiogram of 12 leads in the supine position and an echocardiographic study were conducted with the subjects lying in the left lateral recumbent position, analyzing the transthoracic position, in the parasternal position, the long and short axes, and four apical chambers. “M”, “2D” and “color doppler” modes were used. This ruled out cardiac abnormalities.

Third: A device was placed in the right thigh to evaluate NIRS (Humon Hex, Dynometrics Inc. Cambridge, MA, USA) [17]. Humon Hex weights weighing 32 g, Bluetooth connection, and a 64 MHz processor were used. We used near-infrared spectroscopy to measure hemoglobin saturation in muscles. LEDs emit light into the tissue, at 750 and 850 nm, and detectors measure the intensity of the light propagating through the muscle. The Hex device uses two light sources in NIRS and three photodetectors to measure the intensity of the light that has propagated through the tissue. The photodetectors are located at distances of 1.2, 1.8 and 2.4 cm from the light sources. The acquisition rate was set to 4 Hz. SmO_2_ data were obtained from the Moxzones app (https://moxzones.com/es/). The Humon Hex device was placed at the midpoint of the line that joins, during sitting, the anterior superior iliac spine and the upper edge of the patella. The point was marked with a dermographic pencil. The area was previously been cleaned with hydroalcoholic solution. The device was fixed with the tape provided by the manufacturer. The data of Humon Hex were sent via Bluetooth^®^ to a tablet with the Moxzone^®^ application, which allowed the information to be viewed and collected.

**Figure 2:**
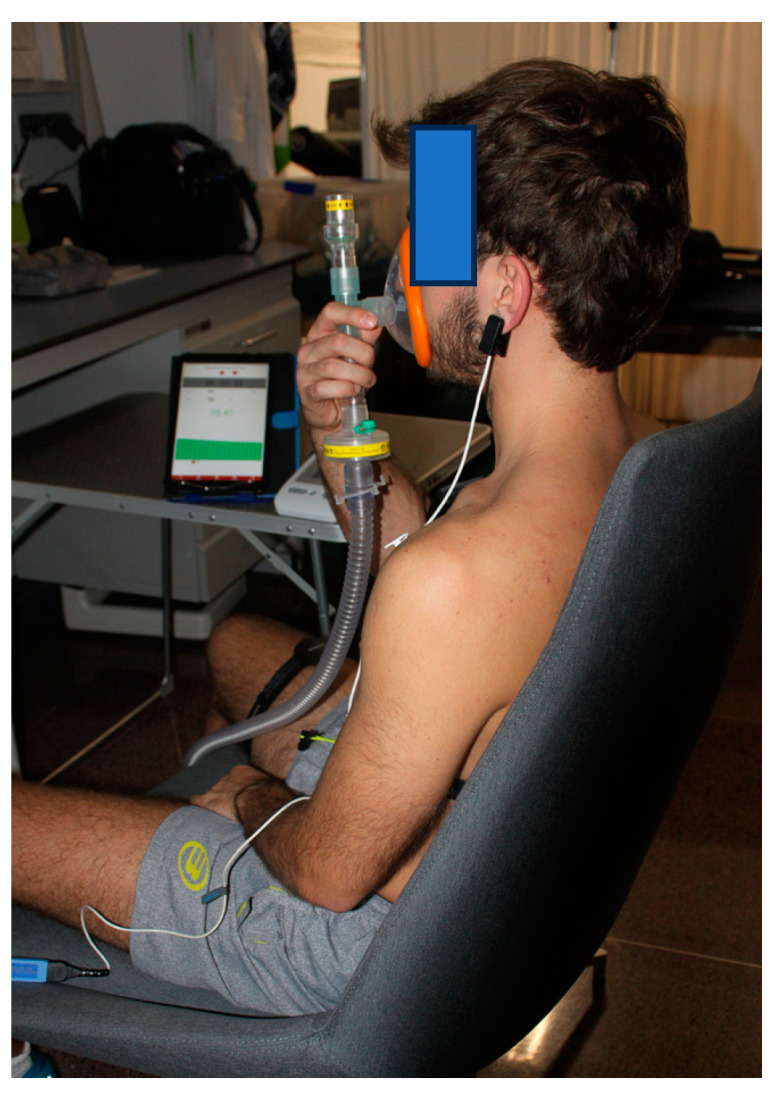
Participant holding the mask with his right hand, with a pulse oximeter on the left earlobe and Humon Hex^®^ on the right thigh with the tablet to collect information on the evolution of SmO_2_.

For the use of the pulse oximeter in the left earlobe (Nonin, Nonin Ear Lobe Clip Sensor, Model 3018LP^®^, Minneapolis, MN, USA), participants were asked to remove the earrings from their earlobe and clean it with hydroalcoholic solution. Subsequently, the pulse oximeter was placed and it was checked that a coherent signal of SpO_2_ and HR was received on the monitor. Using a blood pressure cuff in the right arm (Omrom M3, Omron Healthcare, Kyoto, Japan), blood pressure was measured while seated after a minimum of five minutes of rest. Two measurements were taken, and the lowest values were recorded, and baseline parameters (SmO_2_, SpO_2_, and heart rate (HR)) and systolic and diastolic blood pressure (SBP and DBP) were obtained [3]. Figure 2 shows the placement of the devices.

Fourth: In a seated position, subjects breathed air with 11% FiO_2_, equivalent to that at an altitude of 5050 m, through a mask using the IAltitude training (Ialtitude, Madrid, Spain) device. This simulator allows you to extract an airflow with an FiO_2_ value between 9 and 21%. A regulator can be used to change the percentage of oxygen produced by the machine. It allows a flow volume of 30 L/min at 6500 m and of 90 L/m at 3200 m.

Each participant wore a mask that was previously sterilized, which they adjusted to their face and held with their hands to prevent air leakage. The mask was connected with a flexible tube to the altitude simulator. Between the mask and the tube, there is a system of valves and filters that prevent the return of air and the mixing of exhaled and inhaled air.

The device was calibrated before each use and adapted to the environmental conditions using an analyzer, Handi+ (Maxtec, Salt Lake City, UT, USA). FiO_2_ was then adjusted. On the monitor, the evolution of HR and SpO_2_ was observed, beat by beat. Lines were drawn on axes that mark the values of these variables. A stopwatch indicating the elapsed time was also displayed. When the stipulated time ended or the SpO_2_ drops in relation to the safety value, a warning was issued asking the volunteer to remove the mask. At that time, the values of the measured variables were recorded and the blood pressure was measured.

**Figure 3:**
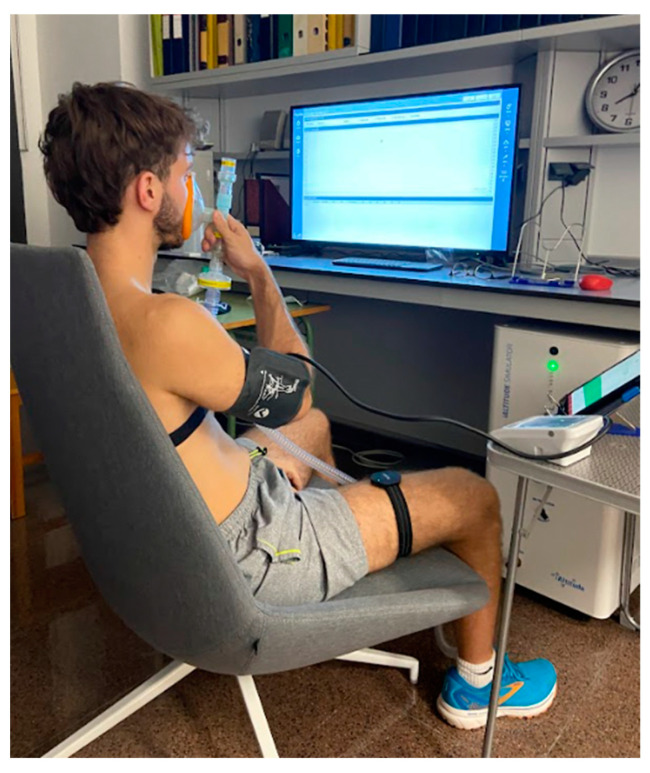
Participant, Ialtitude^®^ device, and HR- and SpO_2_-tracking monitor.

The procedure was ended after 10 min or when an 83% drop in SpO_2_ was observed. Figure 3 shows the tracking of HR and SpO_2_ curves on the monitor.

Fifth: We then obtained the final parameters (hypoxia time, SmO_2_, SpO_2_, HR, SBP and DBP. Subsequently, SmO_2_ and SpO_2_ were obtained after 10 min of breathing under conditions of normoxia.

### 2.4. Variables

The dependent variables were the initial and final values after 10 min of recovery and the differences between the final and initial values of SmO_2_, SpO_2_ and HR. Anthropometric and body composition measurements were independent variables.

### 2.5. Statistical Analysis

Using the SPSS v26^®^ statistical program, the quantitative variables were described using mean values and the standard deviation (SD), and qualitative variables were used as absolute values and percentages. The normality of the variables was checked using the Kolmogorov–Smirnov test, and the normality of the means was tested using the Levene test. An ANOVA test was used for comparison between groups.

Two variables were created per transformation for SmO_2_: (a) post-NHTT—pre-NHTT differences; (b) at 10-min—pre-NHTT differences. To establish the effects of gender and group, an ANCOVA study was carried out for these two variables. Intra-subject comparisons were carried out using the t-paired test, and the effect size was determined using d-Cohen. Correlations were made with Pearson’s *r*-test. It was established that there are significant differences or relationships with a value of *p* < 0.05.

## 3. Results

Table 1 shows the comparison of the characteristics of the participating subjects according to gender.

Table 2 shows the values before and at the end of exposure to the NHTT for the population, as well as the mean and standard deviation of the differences. It is observed that there are significant differences in the three follow-up variables.

In total, 13 of the 37 male participants (35.1%) completed the maximum 10 min of NHTT and 24 did not complete it (64.9%). Of the 17 women, 4 (41.2%) did and 10 (58.8%) did not complete it (Table 3).

After performing ANCOVA for the groups, using SmO_2_ as a dependent variable on the three separate occasions and the differences between the Pre-NHTT SmO_2_ and the other two situations, and using gender as a covariate, the data shown in Table 4 were obtained. It was observed that there was a significant effect of gender on the absolute values of SmO_2_ in all three situations, but not on changes in SmO_2_. The group to which the subjects belonged did not influence the SmO_2_ values. A comparison of SmO_2_ values between subjects who completed the NHTT with those who did not show significant differences in any of the measurements performed is shown (Table 4).

**Figure 4:**
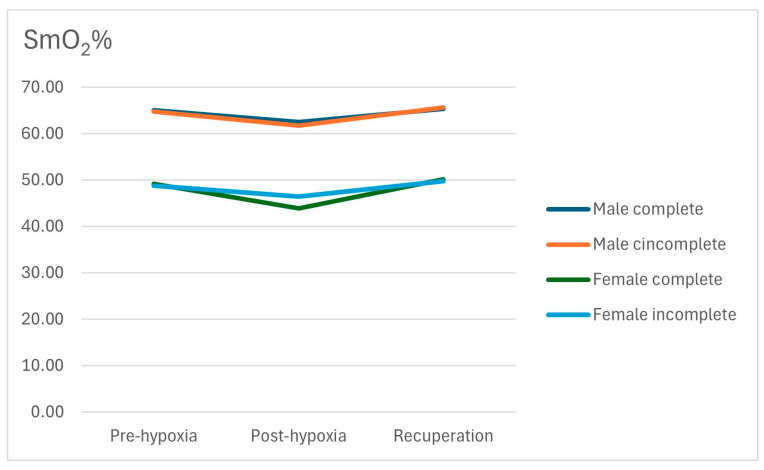
Evolution of SmO_2_ in both groups, separated by gender.

Figure 4 shows the evolution of SmO_2_ percentages in each of the four subgroups: complete male, incomplete male, complete female, and incomplete female.

No correlations were observed between anthropometric variables (height and weight) and body composition variables (percentage of fat mass and percentage of muscle mass) with losses of muscle oxygenation in both absolute and percentage values (*p* > 0.05). (Table 5).

## 4. Discussion

A normobaric hypoxia tolerance test was performed on 54 healthy subjects to establish whether or not the oxygen concentration and the duration of the test are sufficient stimuli to generate changes in muscle oxygenation measured with near-infrared spectroscopy. It was observed that the population could be divided into two subgroups: people who complete the established ten minutes as the maximum duration of the test and those in whom the SpO_2_ decreased below 83% before that time was completed. In both groups, there was a decrease in SmO_2_ that was independent of gender, aged and body composition. During the test, we monitored HR and SpO_2_ using a pulse oximeter in the earlobe and the NIRS device on the right thigh, while other studies, on one hand, obtained the HR through a wireless Polar heart rate monitor [18,19] or a the-lead ECG [20], or, on the other hand, assessed SpO_2_ using a digital pulse oximeter on the right index finger [18]. The continuous evaluation of both variables allows us to analyze their evolution, observing that, while SpO_2_ and SmO_2_ decreased, HR increased in compensation. We believe that continuous recording from a pulse oximeter placed on the earlobe is more comfortable than on the finger, as this allows the subjects to keep their hands free to hold the mask or perform other tasks and does not require the use of the heart rate monitor. The collection of data on muscle oxygenation from the quadriceps does not cause any discomfort for the participant, since when sitting, they are free of all pressure and muscle activity, so it can be ensured that the values obtained are exclusively conditioned by the supply of oxygen. In addition, continuous HR monitoring makes it possible to associate pulsations, in real time, with changes in SpO_2_, identifying changes that suggest the presence of alterations or other stimuli.

We carried out the test at a simulated altitude of 5050 m (FiO_2_ of 0.11) because this was previously used in the planning of this test on performance in hypoxia training. It is an important simulated altitude that is not usually used as a stimulus for training or the search for adaptations, but allows the individual response of each subject to be detected and their potential and pace of adaptation to be established. As a real altitude, it has been used to determine the physiological response to exercise at certain altitudes [21]. Following the pioneers of NH [6], we determined as a criterion the completion of the test a maximum time of 10 min, establishing 83% of SpO_2_ as a safety limit. The recovery of SpO_2_ (values of 98–100%) occurred in less than one minute in all participants breathing ambient air. None of them required supplemental oxygen or any type of recovery maneuver. There were also no changes or discomfort in the two minutes of recovery after the end of the test. We consider these measures to be sufficient to ensure the safety of healthy subjects in this type of test.

The measurement of SmO_2_, using the Humon Hex^®^ device, made it possible to obtain saturation values in both groups of subjects, allowing an observation of the expected changes after the inhalation of oxygen-depleted air. Placement on the vastus lateralis of the right quadriceps is the most common in work performed with this type of technology, regardless of the model or brand used [12,19]. We can see that the decrease in SmO_2_, because of normobaric hypoxia, is like that which occurs with intense exercise, such as a treadmill stress test [17]. Similar effects have been seen in sports such as cycling [18] or medium- and long-distance running [3].

We related SmO_2_ to individual subjects’ response to hypoxia, and we believe that if the effect of physical exercise is included, improvements related to resistance training could be found [22]. In addition, improvements in the oxidative system produce increased subject performance in both sports activities and basic activities of daily living [15]. We believe that the use and monitoring of SmO_2_ during hypoxia training sessions could be an interesting tool to aid in their planning. Training with the help of normobaric hypoxia is performed by combining hypoxia sessions with physical exercise sessions, usually on a cycle ergometer, i.e., breathing oxygen-depleted air while pedaling. In these situations, the information obtained using SmO_2_ can be implemented as another biomarker of muscle adaptation and control of exercise intensity in addition to HR [23], reserving SpO_2_ as a tool to control exercise safety.

The different responses to HN allowed us to study SmO_2_ in two population groups: those who completed the test (reached ten minutes without a significant decrease in SpO_2_) and those who did not. Thus, we had two different types of response: (a) mild hypoxemia maintained over time, and (b) more accentuated hypoxemia, but with less exposure time. We have observed that, from the point of view of muscle oxygenation, there was no difference between these two groups, which makes us think that there must be a grouping factor that considers the time of exposure and the intensity of hypoxemia. Something that could be assimilated to “hypoxia load” would be the product of the hypoxia time due to the value of the maximum decrease in SpO_2_ (100- SpO_2_), and this could be assimilated in such a way that a shorter exposure time would be compensated for by a greater decrease in SpO_2_. An idea used in training is higher altitude with less time. Although this, in part, has already been described by Bassovitch and Serebrovskaya [6], it requires further studies to adapt it to SmO_2_.

The population of our study, like that of Camacho-Cardenosa [23], was composed of women and men, unlike most studies on normobaric hypoxia that have been conducted only with men [24,25]. We observed that, overall, the behavior of HR and SpO_2_ was similar in both genders with no significant differences between them in the final initial values. On the other hand, as in previous studies, SmO_2_ initial determinations showed significantly higher values in males, both at the beginning and at the end of the test, but with a similar rate of decline in both groups [26].

In a study by Hobbins et al. [27], it was found that the response to normobaric hypoxia is not affected by body composition and anthropometric variables. We also did not see any effects of these variables on SmO_2_. On the other hand, age was not a determining factor in the results either.

The decrease in SmO_2_ is related to the onset of fatigue, which in turn is related to the intensity of exercise and the loss of oxidative capacity; therefore, the acute response of SmO_2_ to NH is like that in intense physical exercise [10]. The decrease in SmO_2_ has been studied during maximal exercise in the laboratory, observing that it usually occurs in different phases of longer or shorter duration depending on the intensity of the exercise [28], and this has also been related to oxygen consumption and ventilatory thresholds [29]. With our work, the association of SmO_2_ with exposure to hypoxia may serve to support new studies in which it is applied to training at a certain altitude. The muscular response to exercise may depend on the intensity of the exercise and the muscle oxygenation associated with hypoxemia.

An interesting point to be studied is how the decrease in SmO_2_ occurs due to hypoxia, establishing whether it is linear or forms curves like those that appear with strenuous exercise [30]. An aspect of interest from our work is to analyze how this curve behaves during recovery. Another contribution of this study, considering that NH is an emerging therapy, is to accept that the measurement of SmO_2_ can be used to monitor the effectiveness of NH, analyzing its effect on peripheral muscles. Monitoring the evolution of SmO_2_ is a simple way to understand and determine the role of the different types of hemoglobin involved in muscle oxygenation and physical exercise [16]. Therefore, it could be used as a biological marker of the effect of normobaric hypoxia both in the training of athletes and as an adjuvant to the treatment of chronic pathologies [31].

One of the limitations of our work is that its conclusions can only be attributed to the population we analyzed (healthy and active subjects) but not to people with pathologies or with a high level of training. It was carried out with a concentration of O_2_ and within a certain time, so the results cannot be extrapolated to conditions with other concentrations of O_2_, or to the effect of a training program. However, they open up an interesting avenue to follow.

We conclude that performing a hypoxia tolerance test at a 5050 m simulated altitude caused a decrease in quadriceps SmO_2_ in all exposed subjects, both in the people who completed the test, i.e., the group with “longer and less intensity of hypoxia”, and in the group of those who reached SpO_2_ values of 83% and, therefore, did not complete the test, and who defined as “less time and more hypoxia”.

## Figures and Tables

**Table 1:** Gender differences in general characteristics (37 men and 17 women).

	Gender	Mean	SD	ANOVA (F)	*p* Value
Aged (years)	Men	30.9	10.5	1.756	0.191
	Women	27.1	8.4		
Weekly Physical Exercise (Hours)	Men	7.24	4.60	4.535	0.038
	Women	4.65	2.94		
Height (cm)	Men	177.14	6.74	35.877	<0.000
	Women	165.18	6.97		
Body Weight (Kg)	Men	77.64	9.57	29.239	<0.001
	Women	62.58	9.34		
BMI (Kg/m^2^)	Men	24.72	2.24	7.231	0.010
	Women	22.89	2.50		
Fat percentage (%)	Men	19.06	5.21	30.702	<0.001
	Women	28.22	6.52		
Muscle Percentage (%)	Men	42.50	5.39	8.501	0.005
	Women	37.91	5.33		
Waist circumference (cm)	Men	80.77	6.53	22.317	<0.001
	Women	71.49	7.07		
Hip circumference (cm)	Men	95.74	6.78	0.079	0.780
	Women	95.21	5.59		
SBP (mmHg)	Men	129.08	13.71	16.047	<0.001
	Women	113.53	12.15		
DBP (mmHg)	Men	80.73	9.04	1.242	0.270
	Women	77.65	10.30		

SD = standard deviation. SBP = systolic blood pressure. DBP = diastolic blood pressure.

**Table 2:** Pre- and post-NHTT values of saturations and HR (*n* = 54).

		Mean	SD	Mean Dif	SD Dif	*t*-Paired	*p*-Value	d Cohen
SpO_2_ (%)	Pre	99.17	1.22	14.46	4.61	23.043	<0.000	3.103
	Post	84.70	4.68					
SmO_2_ (%)	Pre	59.85	12.99	3.11	3.52	6.502	<0.000	0.803
	Post	56.74	13.00					
HR (bpm)	Pre	73.83	13.99	−6.65	9.48	−5.156	<0.000	−0.685
	Post	80.48	12.79					

dif = difference; SD: standard deviation; SpO_2_ = peripheral oxygen saturation; SmO_2_ = muscle oxygen saturation; HR = heart rate; bpm = beats per minute.

**Table 3:** Descriptive values of the different moments of SmO_2_ according to whether or not subjects had performed the complete test or not, separated by gender and all subjects.

	SmO_2_ (%)	Group	*n*	Mean	SD
Men (n = 37)	Pre	Incomplete	24	64.75	10.22
		Complete	13	65.08	5.74
	Post	Incomplete	24	61.71	10.23
		Complete	13	62.46	5.92
	10 min	Incomplete	24	65.67	8.83
		Complete	13	65.31	6.22
Women (n = 17)	Pre	Incomplete	10	48.80	14.09
		Complete	7	49.14	15.31
	Post	Incomplete	10	46.40	10.50
		Complete	7	43.86	17.74
	10 min	Incomplete	10	49.80	9.59
		Complete	7	50.14	17.85
All subjects (n = 54)	Pre	Incomplete	34	60.06	13.46
		Complete	20	59.50	12.48
	Post	Incomplete	34	57.21	12.38
		Complete	20	55.95	14.30
	10 min	Incomplete	34	61.00	11.54
		Complete	20	60.00	13.42

Pre = prior to the tolerance test. Post = after the tolerance test. SD = standard deviation.

**Table 4:** ANCOVA. SmO_2_ (*n* = 54).

SmO_2_	Gender (*p* Value)	Group (*p* Value)
Pre NHTT	<0.000	0.914
Post NHTT	<0.000	0.913
10 min NHTT	<0.000	0.963
Post—Pre NHTT	0.532	0.513
10 min—PreNHTT	0.772	0.696

NHTT = normobaric hypoxia tolerance test. Pre = prior to the tolerance test. Post = after the tolerance test.

**Table 5:** Correlations between anthropometric and body composition variables with changes in SmO_2_ (differences between post-NHTT and pre-NHTT).

	Men		Women	
	*r* Pearson	*p* Value	*r* Pearson	*p* Value
Height (cm)	0.193	0.252	0.331	0.194
Body Weight (Kg)	0.095	0.578	0.252	0.330
Muscle mass (Kg)	−0.199	0.237	0.290	0.259
Fat mass (Kg)	0.096	0.572	0.213	0.413
BMI (Kg/m^2^)	−0.037	0.826	0.104	0.691
Fat percentage (%)	0.060	0.726	0.076	0.772
Muscle Percentage (%)	−0.318	0.055	0.142	0.586
Waist circumference (cm)	0.112	0.509	0.242	0.349

## Data Availability

The data can be requested by e-mail from the corresponding author.
